# Sudden-Onset Severe Thrombocytopenia Secondary to Trimethoprim-Sulfamethoxazole

**DOI:** 10.7759/cureus.91830

**Published:** 2025-09-08

**Authors:** Jonathan Spadafora, Philip Spadafora, James Spadafora, Jonathan Starcke, Philip F Spadafora

**Affiliations:** 1 College of Osteopathic Medicine, New York Institute of Technology, Old Westbury, USA; 2 Internal Medicine, NYU Langone Hospital – Long Island, Mineola, USA

**Keywords:** drug‑induced immune thrombocytopenia, petechiae, platelet transfusion, sudden-onset thrombocytopenia, tmp‑smx (trimethoprim‑sulfamethoxazole)

## Abstract

Drug-induced immune thrombocytopenia (DITP) is a rare but life-threatening condition characterized by a sudden and serious drop in the number of platelets from drug-dependent antibodies against platelet glycoproteins. We report the case of a 57-year-old man who developed severe thrombocytopenia and mucocutaneous bleeding following a short course of trimethoprim-sulfamethoxazole (TMP-SMX) for presumed tick-borne disease. The patient experienced bleeding gums, pinpoint rashes, bruising, and extreme fatigue. The laboratory tests indicated a severely low platelet count of 1 × 1.0 × 10³/µL combined with a high immature platelet fraction (IPF). Workup for both infection and autoimmune disorders would be negative, along with the absence of intracranial hemorrhage demonstrated on imaging. The timeline and clinical picture suggested that TMP-SMX was the most likely culprit. The patient was placed on supportive care, including platelet transfusion and re-start of doxycycline after discontinuation of the drug. His platelet count began to rise, and his symptoms improved; he was discharged without issues. By presenting this instance, we place high importance on early recognition and immediate withdrawal of the offending agent to prevent life-threatening bleeding. Though confirmatory testing of antibodies is available, clinical diagnosis remains the gold standard due to delays in testing and availability.

## Introduction

Thrombocytopenia refers to a condition where the blood platelet count drops below 150 x 10³/µL. Like most things hematologic, there are many causes that can lead to these conditions. These include infections, autoimmune disease, malignancy, and drug reaction, among others. Drug-induced immune thrombocytopenia (DITP) is defined as an uncommon, yet life-threatening condition that is caused by the sudden onset of severe platelet destruction [1]. Patients with DITP usually develop signs and symptoms suddenly. There may be mucocutaneous bleeding manifestations, which include petechiae, ecchymoses, and mucosal hemorrhage. In some cases, the platelet count may decrease to less than 10,000/μL, indicating severe thrombocytopenia. This greatly increases the risk of spontaneous bleeding, which can be life-threatening.

An excess of 100 drugs is thought to trigger DITP. The most implicated agents are antibiotics such as vancomycin, beta-lactams, and trimethoprim-sulfamethoxazole (TMP-SMX) [2]. TMP-SMX is often given for a wide range of antimicrobial therapy and is generally well tolerated. However, there have been rare instances of immune-mediated thrombocytopenia with its use [3]. The mechanism in play is thought to create drug-dependent antibodies that bind to platelet surface glycoproteins only in the presence of TMP-SMX, which accelerates platelet clearance [4].

The diagnosis of DITP is mainly based on clinical factors. It relies on the timing since drug exposure and the onset of thrombocytopenia. Other causes, such as disseminated intravascular coagulation and primary ITP, need to be excluded. The platelet count should increase rapidly upon withdrawal of the agent. The laboratory tests of drug-dependent platelet antibodies may help to confirm the diagnosis but are not widely available and may have limited clinical value due to delayed results [1].

This is a case of a previously healthy adult who developed severe TMP-SMX-induced immune thrombocytopenia presenting with bleeding and rash. What makes this case distinctive is the diagnostic challenge created by a recent tick bite, which initially raised suspicion for a tick-borne infection rather than a drug reaction. In addition, the markedly elevated immature platelet fraction (IPF) provided supportive evidence of peripheral platelet destruction with preserved bone marrow function, a finding that is not commonly reported in prior TMP-SMX-related cases. This case, therefore, emphasizes the importance of including DITP in the differential diagnosis of acute thrombocytopenia in patients recently exposed to TMP-SMX, even when an infectious etiology is strongly suspected. It further highlights the critical role of prompt drug discontinuation in facilitating recovery and preventing serious hemorrhagic complications.

## Case presentation

**Figure 1:**
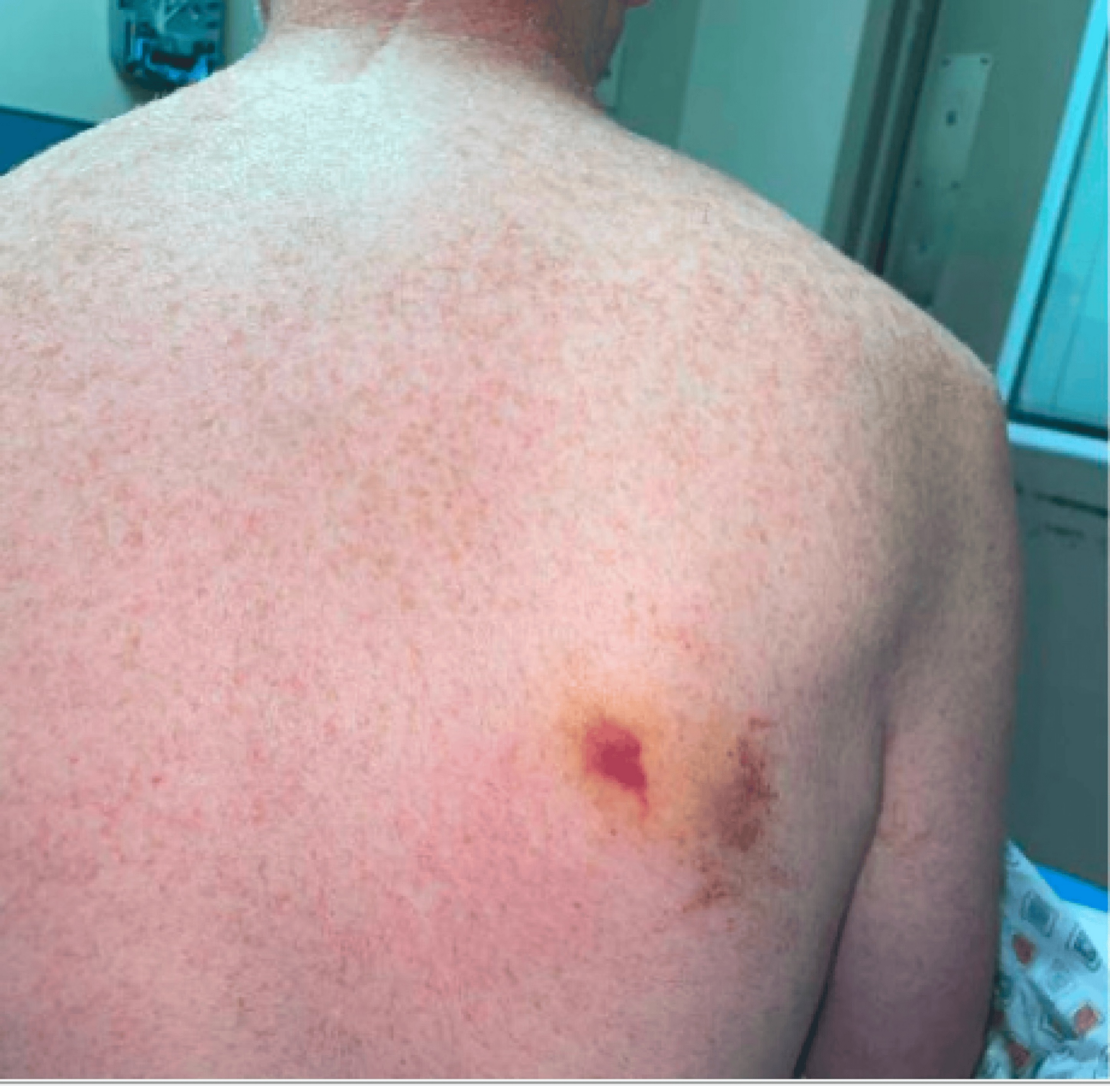
Ecchymotic lesion from tick bite on the patient’s right middle back

**Figure 2:**
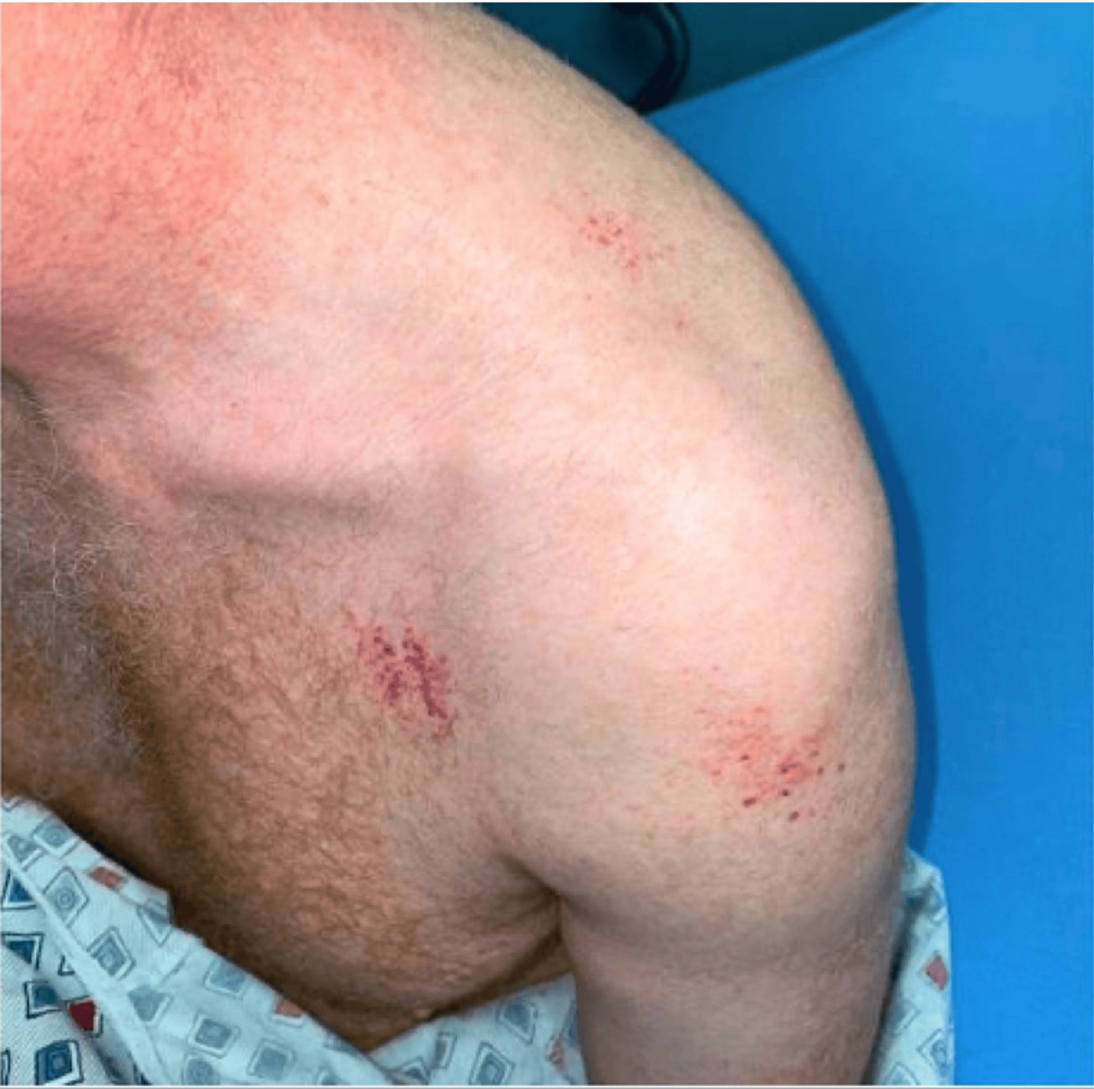
Pinpoint petechiae on the bilateral shoulder and chest

The patient is a 57-year-old male with a past medical history of hyperlipidemia and cervical spinal stenosis who presented to the emergency department with mild epistaxis, severe fatigue, gingival bleeding, petechial rash on both shoulders, and an ecchymotic lesion on his scapula (Figures 1, 2). Five days prior to presentation, he sustained a tick bite and developed a localized erythematous rash at the bite site without systemic symptoms, for which doxycycline prophylaxis was initiated by his primary care physician. The localized erythematous rash resolved with doxycycline; however, three days prior to presentation, shortly after starting TMP-SMX, when the initial rash persisted, he developed new-onset petechiae, gingival bleeding, and worsening fatigue. He reported taking approximately six doses of TMP-SMX over the course of three days before discontinuation was advised due to these symptoms. He denied any prior similar bleeding or rash and had no family history of hematologic disorders.

Upon arrival at the emergency department, physical examination was pertinent for bilateral mild petechial, non-tender, non-pruritic rash on the shoulders and ecchymotic, pruritic, non-tender lesions on the right middle back near the scapula. The remainder of the physical examination was unremarkable. Initial vital signs included a blood pressure of 165/105 mmHg, heart rate of 89, temperature of 98.6°F, respiratory rate of 18 bpm, and oxygen saturation of 98% on room air. A complete blood count was ordered and showed severe thrombocytopenia of 1.0 × 10³/µL (normal 150-400 x 10³/µL). IPF was 25.2% (relative), monocytes accounted for 16%, and atypical lymphocytes for 2% of the differential count. A basic metabolic panel showed potassium of 5.7 mEQ/L. Hemolysis labs, including LDH, bilirubin, haptoglobin, and direct antiglobulin test (DAT/Coombs), were within normal limits, consistent with isolated thrombocytopenia rather than hemolysis. With an initial concern of tick-borne illness, serologic testing was ordered for multiple microbes such as *Borrelia burgdorferi*, *Rickettsia*, *Ehrlichia chaffeensis*, *Anaplasma phagocytophilum*, and *Babesia microti*. Additional tests, folate, polymerase chain reaction (PCR) for COVID and influenza, C-reactive protein (CRP), erythrocyte sedimentation rate (ESR), magnesium, alkaline phosphatase (ALP), aspartate transaminase (AST), alanine transaminase (ALT), and bilirubin were all within normal limits, helping narrow down this diagnosis.

**Figure 3:**
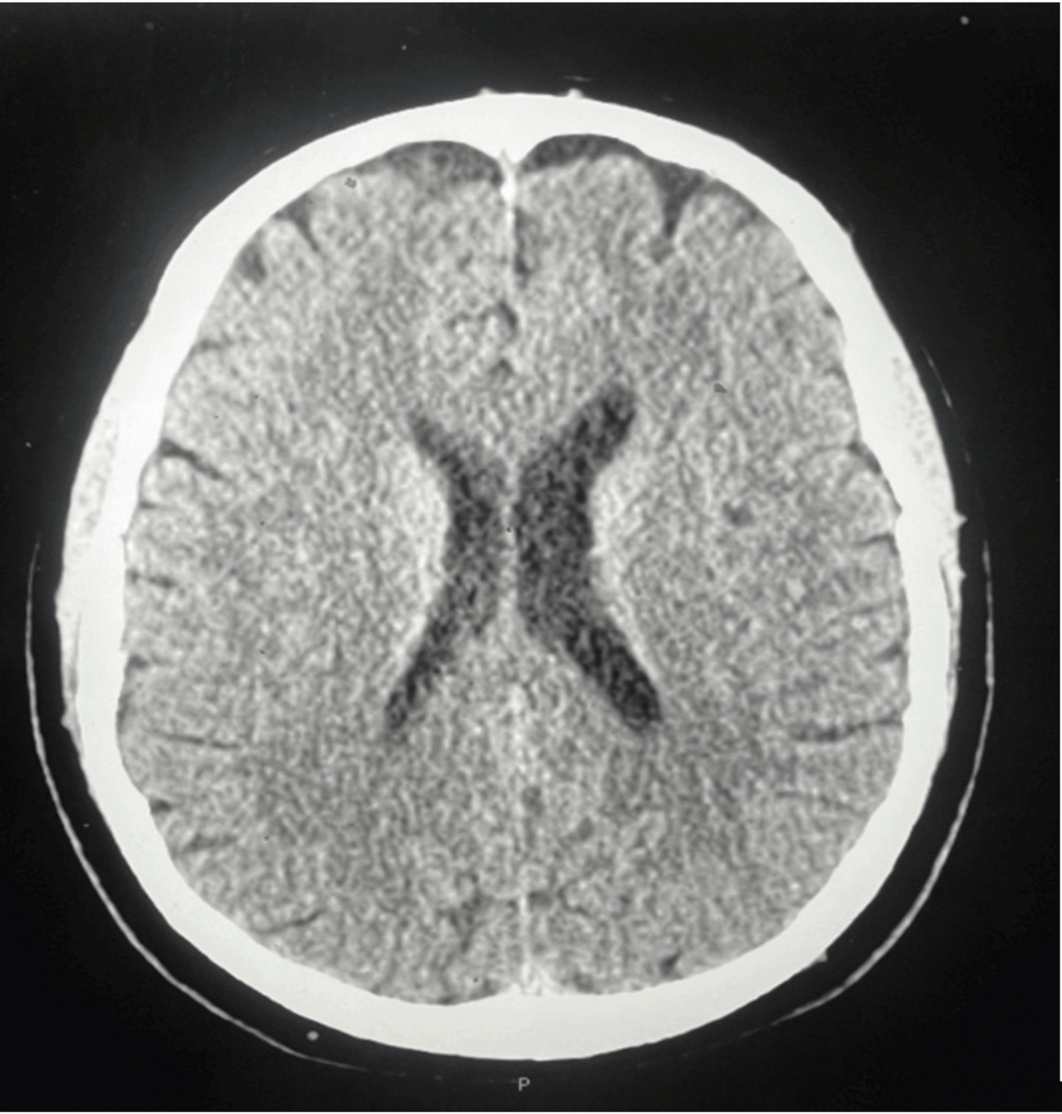
CT scan of the patient's brain No acute intracranial abnormalities

These ultimately were negative, ruling out this differential. Additional tests were ordered, a CT scan of the brain without IV contrast to which showed no acute intracranial abnormalities, as shown in Figure 3. An electrocardiogram was performed to monitor any cardiac issues; the results showed a normal sinus rhythm with no other abnormalities.

Given the presence of the patient’s symptoms with the differential being DITP and a platelet count of 1 × 10³/L, the patient received an urgent transfusion of two units of platelets and restarted on 100 mg doxycycline 2x per day for 10 days, as well as discontinuation of Bactrim. Mild hyperkalemia was also seen on day 2, which is also a known but less common side effect of Bactrim, as demonstrated by Table 1. Upon transfusion and supportive care, platelets went up to 75 × 10³/µL when measured 60 minutes later. Following hospitalization on day 2, platelet levels continued to rise, now at 80 × 10³/µL, and all other values fell within normal parameters. The hematology and infectious disease teams were consulted, and the patient was able to be discharged without any complications following a one-day stay. After discharge, four days later, the platelet count returned to within normal range, i.e., 210 × 10³/µL, as shown by Table 2. 

**Table 1:** Renal functional panel of the patient on days 2 and 3 eGFR: estimated glomerular filtration rate

Renal functional panel	Normal blood values	Day 2	Day 3
Sodium	136-145	137 mEQ/L	139 mEQ/L
Potassium	3.5-5.1	5.7 mEQ/L (HIGH)	4.1 mEQ/L
Chloride	98-107	105 mEQ/L	106 mEQ/L
Carbon dioxide	22-29	22 mEQ/L	21 mEQ/L (LOW)
Blood urea nitrogen	8-26	13 mg/dL	12 mg/dL
Glucose	70-100	93 mg/dL	88 mg/dL
Creatinine	0.7-1.3	1.11 mg/dL	0.93 mg/dL
Calcium	8.4-10.4	9.2 mg/dL	9.1 mg/dL
Anion gap	6-14	10 mEQ/L	12 mEQ/L
Albumin	3.5-5.2	N/A	4.0 g/dL
Phosphorus inorganic	2.3-4.7	N/A	3.0 mg/dL
eGFR	>60	77.9 mL/min	96.4 mL/min

**Table 2:** Complete blood counts of the patient on day 1 and days 4, 5, and 6 WBC: white blood cell, RBC: red blood cell, HB: hemoglobin, HCT: hematocrit, MCV: mean corpuscular volume, MCH: mean corpuscular hemoglobin, MCHC: mean corpuscular hemoglobin concentration, PLT: platelets, IPF: immature platelet fraction

Complete blood count	Normal lab values	Day 1	Day 2 (hospital)	Day 2 (post infusion)	Day 3	Day 6
WBC count	3.4-10.8	5.8 x 10³/µL	5.6 x 10³/µL	6.5 x 10³/µL	5.0 x 10³/µL	7.4 x 10³/µL
RBC count	4.14-5.8	4.89 x 10⁶/µL	5.03 x 10⁶/µL	4.81 x 10⁶/µL	4.65 x 10⁶/µL	4.89 x 10⁶/µL
HB	13.0-17.0	15.1 g/dL	14.8 g/dL	14.2 g/dL	14.0 g/dL	14.7 g/dL
HCT	37.5-51.0	44%	44.9%	43.1%	41.3%	44.8%
MCV	79.0-97.0	90 fL	89.3 fL	89.6 fL	88.8 fL	91.6 fL
MCH	26.6-33.0	30.9 pg	29.4 pg	29.5 pg	30.1 pg	30.1 pg
MCHC	31.5-35.7	34.3 g/dL	33.0 g/dL	32.9 g/dL	33.9 g/dL	32.8 g/dL
RDW	11.5-15.4	12.8%	13.5%	13.3%	13.7%	12.7%
PLT count	150-400	1 x 10³/µL (low)	8 x 10³/µL (low)	75 x 10³/µL (low)	80 x 10³/µL (low)	210 x 10³/µL
IPF	1.0-7.0	N/A	25.2 (high)	6.0%	6.9 %	N/A
Monocytes	0.1-0.9	11.7%	16% (high)	13% (high)	12%	N/A
Atypical lymphocytes	0	N/A	2% (high)	0%	0%	N/A

Although thrombocytopenia is a known manifestation of several tick-borne infections, the clinical course in this patient was not consistent with an infection-related process. The absence of systemic features such as fever, leukopenia, or transaminitis, together with negative serologic testing, made an infectious etiology less likely. By contrast, the abrupt onset of profound thrombocytopenia within three days of TMP-SMX initiation, followed by rapid platelet recovery after drug discontinuation, strongly supports a drug-induced mechanism. The close temporal association between TMP-SMX exposure and platelet count trajectory was therefore the key factor in distinguishing DITP from infection-related thrombocytopenia in this case.

## Discussion

DITP is a unique immune-mediated response; several pathogenic mechanisms for DITP have been proposed, including hapten formation, autoantibody, neoepitope, drug-specific antibodies, and quinine-type drug mechanisms [5]. Drug-dependent antibodies are uncommon antibodies that bind tightly to specific sites on platelet surface glycoproteins in the presence of the sensitizing drug. Platelet-targeting drugs attach to platelets and inhibit platelet function. These drugs often bind non-covalently and reversibly to platelets, usually to sites on GP IIb-IIIa and/or GP Ib-V-IX but also to the antibody itself [6]. Although unlikely to occur, DITP has been shown to affect about 10 people per million with no definitive risk factors that would predispose patients to this condition [7].

TMP-SMX is a broad-spectrum antibiotic used to treat many infections; that being said, it has gained traction over the past few years and is being prescribed more often due to its efficacy and ease of use. TMP-SMX is usually well tolerated and has a mild side effect profile, such as rashes and gastrointestinal upset, with some rare reports such as hyperkalemia [8]. TMP-SMX is also known to cause DITP, a rare but potentially fatal unwanted side effect [9]. 

Physical examination should be normal except for bleeding, which may be minimal bruising, whereas others experience serious bleeding, which may include gastrointestinal hemorrhage (GI), extensive skin and mucosal hemorrhage, or intracranial hemorrhage (ICH). In younger individuals, splenomegaly may be present, but there should be an absence of systemic symptoms such as fever, weight loss, or lymphadenopathy. On peripheral blood smear, there should be isolated thrombocytopenia with a normal complete blood count [10]. A platelet count under 10,000 would put the patient at a severe risk for spontaneous bleeding and intracranial hemorrhage with a high incidence of fatality [11].

The first-line treatment is to discontinue offending agents. It is common practice to administer corticosteroids, but whether they are beneficial in these kinds of pathological instances is not proven. Patients who are severely thrombocytopenic benefit from platelet transfusions to lower the risk for intracranial hemorrhage. It is rare, but when bleeding persists for weeks, treatment involves administration of IVIG and possible plasma exchange [2]. Aside from clinical diagnosis, drug-dependent antiplatelet antibodies are important to confirm the etiology of DITP. These tests are not readily available for providers and take a considerable amount of time to get results, making them not widely used or useful, as this is a diagnosis that needs to be acted on immediately. Moreover, in some cases, these drug assays can be negative in patients who do have DITP [6,12]. In this case, the patient improved rapidly with supportive care alone, without corticosteroids or IVIG, highlighting the reversibility of DITP when promptly recognized. In addition, the markedly elevated IPF provided strong evidence of peripheral platelet destruction, further linking the discussion directly to the clinical findings. 

## Conclusions

DITP can be a dangerous and under-recognized drug reaction. This case illustrates the necessity for clinical awareness of sudden-onset thrombocytopenia, especially after exposure to potentially high-risk drugs such as TMP-SMX. In patients with acute thrombocytopenia following TMP-SMX exposure, DITP should be promptly considered, although infection-related thrombocytopenia must also remain in the differential. Although DITP is quite rare, it may progress rapidly and cause significant bleeding, requiring prompt recognition and management. The most important treatment is stopping the offending agent, which often leads to a good response in platelet counts. While confirmatory testing with drug-dependent antibody assays may assist in the diagnosis, unavailability in acute scenarios should not delay empiric management. This case emphasizes the importance of complete medication history taking and the need to include DITP in the differential diagnosis of acute thrombocytopenia. Clinicians being more aware may help in earlier diagnosis, prevention of unnecessary interventions, and improved outcomes in patients.

Although the patient presented after a recent tick bite, which initially raised concern for an infection-related etiology, several factors supported TMP-SMX-induced thrombocytopenia as the most likely diagnosis. Tick-borne infections are well recognized to cause thrombocytopenia; however, the absence of systemic features such as fever, negative infectious serologies, and, most importantly, the close temporal relationship between TMP-SMX initiation, abrupt platelet decline, and rapid recovery following drug discontinuation all argue against an infectious process. This case, therefore, illustrates not only the importance of considering DITP in patients exposed to TMP-SMX but also the critical role of careful timeline analysis in distinguishing drug-induced from infection-related causes of acute thrombocytopenia. In addition, the elevated IPF in this patient further supports peripheral platelet destruction and reinforces the diagnosis of DITP.
